# Human embryonic stem cell (hES) derived dendritic cells are functionally normal and are susceptible to HIV-1 infection

**DOI:** 10.1186/1742-6405-5-1

**Published:** 2008-01-23

**Authors:** Sriram Bandi, Ramesh Akkina

**Affiliations:** 1 Department of Microbiology, Immunology and Pathology, Colorado State University, Fort Collins, Colorado 80523, USA; 2 Department of Medicine, Marion Bessin Liver Research Center and Center for Human Embryonic Stem Cell Research, Albert Einstein College of Medicine, Bronx, New York 10461, USA

## Abstract

**Background:**

Human embryonic stem (hES) cells hold considerable promise for cell replacement and gene therapies. Their remarkable properties of pluripotency, self-renewal, and tractability for genetic modification potentially allows for the production of sizeable quantities of therapeutic cells of the hematopoietic lineage. Dendritic cells (DC) arise from CD34+ hematopoietic progenitor cells (HPCs) and are important in many innate and adaptive immune functions. With respect to HIV-1 infection, DCs play an important role in the efficient capture and transfer of the virus to susceptible cells. With an aim of generating DCs from a renewable source for HIV-1 studies, here we evaluated the capacity of hES cell derived CD34+ cells to give rise to DCs which can support HIV-1 infection.

**Results:**

Undifferentiated hES cells were cultured on S17 mouse bone marrow stromal cell layers to derive CD34+ HPCs which were subsequently grown in specific cytokine differentiation media to promote the development of DCs. The hES derived DCs (hES-DC) were subjected to phenotypic and functional analyses and compared with DCs derived from fetal liver CD34+ HPC (FL-DC). The mature hES-DCs displayed typical DC morphology consisting of veiled stellate cells. The hES-DCs also displayed characteristic phenotypic surface markers CD1a, HLA-DR, B7.1, B7.2, and DC-SIGN. The hES-DCs were found to be capable of antigen uptake and stimulating naïve allogeneic CD4+ T cells in a mixed leukocyte reaction assay. Furthermore, the hES-DCs supported productive HIV-1 viral infection akin to standard DCs.

**Conclusion:**

Phenotypically normal and functionally competent DCs that support HIV-1 infection can be derived from hES cells. hES-DCs can now be exploited in applied immunology and HIV-1 infection studies. Using gene therapy approaches, it is now possible to generate HIV-1 resistant DCs from anti-HIV gene transduced hES-CD34+ hematopoietic progenitor cells.

## Background

Human embryonic stem (hES) cells are endowed with pluripotential and self-renewal properties [1,1]. In addition, they are tractable for stable genetic modification. These attributes qualify them as potential candidates to derive an unlimited supply of any cell type for transplantation, gene therapy, drug screening and functional genomic applications. A number of previous studies have demonstrated the ability of hES cells to differentiate into a myriad of cell types that include neurons, hematopoietic cells, cardiomyocytes, and insulin-secreting cells, to name a few [3-9]. Many new studies are currently directed towards expanding the use of hES cells for novel applications.

In this regard, the ability to generate cells of the hematopoietic system has considerable potential in several areas of clinical and experimental medicine as they can reconstitute the entire blood system and can serve as primary targets in gene therapy in treating infectious diseases such as AIDS and inherited diseases [9,10]. Given the present lack of effective vaccines and the ineffectiveness of drug based therapies for a complete cure with regard to HIV/AIDS, new and innovative approaches are essential [10,11]. Gene therapy through intracellular immunization offers a promising alternative approach and possible supplement to current HAART therapy. A primary goal of many ongoing studies is to introduce an effective anti-HIV gene into hematopoietic progenitor cells [11]. As these cells possess the ability to self-renew, they have the potential to continually produce HIV resistant T cells, macrophages, and dendritic cells in the body thus providing long term immune reconstitution. These approaches use CD34+ hematopoietic stem cells for anti-HIV gene transduction via integrating viral vectors such as lentiviral vectors. Current sources of CD34+ cells are restricted to human umbilical cord blood (CB), adult bone marrow (BM), mobilized peripheral blood, (MPB), and fetal liver [11]. hES cells are a good viable alternative for the generation of an unlimited supply of CD34+ cells thus paving the way for utilization of these cells for hematopoietic cell therapy [9]. Recently we demonstrated derivation of phenotypically and functionally normal macrophages from hES-CD34+ cells and established that they could support HIV-1 infection. These studies laid the ground work for utilizing hES-CD34+ cells in HIV research and for testing anti-HIV genes in a gene/cell therapy setting [10].

Similar to monocytes/macrophages, dendritic cells (DCs) also originate from hematopoietic progenitor cells and spread via the bloodstream and lymphatics [12,13]. They are found in almost every organ as sentinels of the immune system. In innate immunity, DCs function via type-1 interferon activation of both macrophges and NK cells. In adaptive immunity, DCs constitute the most powerful antigen presenting cells (APCs) that prime naïve T lymphocytes and sensitize cytotoxic T lymphocytes to the antigens they present [13]. Thus, efficient generation of these cells from renewable sources such as hES cells would have great potential for immunotherapy applications. However in HIV-1 infection, in addition to being infected and functionally compromised, paradoxically they are also culprits in the efficient transfer of the virus to susceptible cells [14]. Thus in gene therapy applications for HIV infections they are also among principal cells that need to be protected. For such efforts to proceed further, it is important to evaluate if hES derived DCs are functionally normal and support HIV-1 infection. As a first step towards this goal, here we show that hES-CD34+ cells can give rise to normal DCs which are capable of supporting HIV-1 infection.

## Results

### Hematopoietic differentiation of human ES cells and derivation of dendritic cells

**Figure 1:**
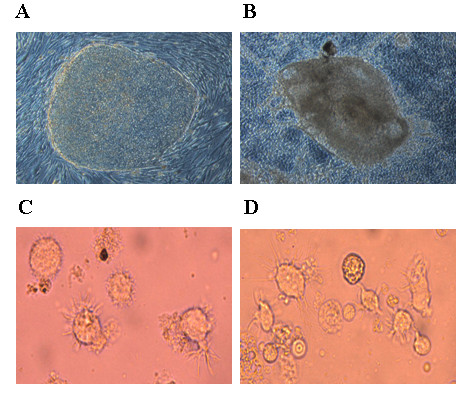
**Derivation of DCs from hES cells**: Undifferentiated hES cells were cocultured with S17 mouse stromal cells to derive cystic bodies. Later, purified CD34+ cells derived from cystic bodies and fetal liver were cultured in cytokine media to derive DCs as described in Methods. A and B, representative hES colony and an cystic body respectively. C and D, morphology of DCs differentiated from hES and FL derived CD34+ cells.

**Figure 2:**
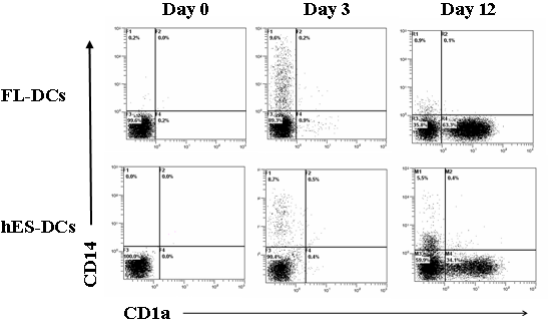
**FACS analysis of differentiating DCs from hES and FL CD34+ cells**: CD34+ cells were cultured in cytokine media and analyzed by FACS for CD14 and CD1a markers at different days by staining with CD1a-PECY5 and CD14-PE conjugated antibodies. Dot plots are representative of triplicate experiments.

hES cell line H1 was propagated as undifferentiated cells by co-culture on mitomycin treated MEF feeder layers. Consistent with previous studies, cells cultured in this manner grew as tightly packed colonies (Figure 1A). To promote hematopoietic differentiation, the H1 cell colonies were cocultured with irradiated S17 cells [10]. After 4–7 days in culture, the H1 cells differentiated into cystic bodies (Figure 1B) which were allowed to further expand for 14–17 days. FACS analysis of single cell suspensions of the differentiated cells showed the development of CD34+ cells (range 7% to 15%, data not shown). Purified CD34+ cells were later cultured in dendritic cell differentiation media in parallel with CD34+ cells from fetal liver. Cell morphology and phenotypic properties were monitored periodically. By day 12, the differentiating cells showed a characteristic veiled or dendritic appearance with numerous cytoplasmic extensions indicative of DC development (Figure 1C). FL-DCs and hES-DCs were found to be morphologically similar as seen in the phase-contrast images (Fig 1C and 1D). We also looked for the expression markers during differentiation and figure 2 illustrates CD1a and CD14 expression at days 0, 3, and 12. At day 0, CD34+ cells expressed neither CD1a nor CD14. By day 3, two cell populations expressing either CD1a or CD14 could be seen with the CD14 single positive population being the majority. With hES CD34+ cells, by day 12, 34% of the cells differentiated into CD1a expressing DCs. The overall yield of CD1a+ cells prepared in this manner ranged from 8–34% for hESC-DCs and 20–64% for FL-DCs.

### hES derived DCs (hES-DC) express normal DC surface markers

**Figure 3:**
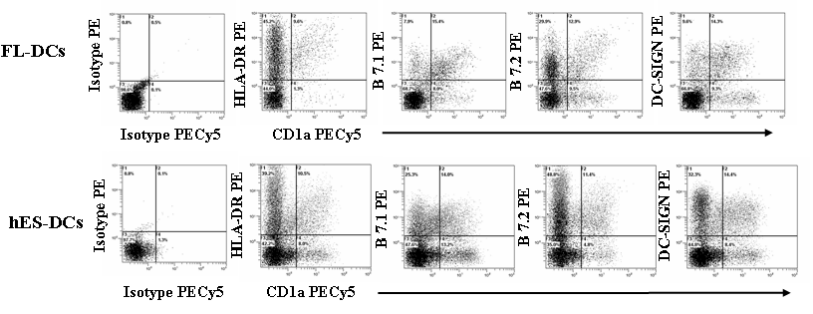
**Phenotypic analysis of hES-DCs and FL-DCs**: hES-DCs and FL-DCs were stained with antibodies CD1a-PECY5, HLA-DR-PE, B7.1-PE, B7.2-PE, and DC-SIGN-PE. Expression of these respective markers was analyzed by FACS. Percent positive cells are indicated in respective plots for each of the cell surface markers. The isotype controls are shown in the left panel. Data is representative of triplicate experiments.

The mature DCs were generated from hES cells by culturing for 12 days in media containing the growth factors and cytokines as mentioned above. Following differentiation, hES-DCs were analyzed for expression of the human class II antigen presenting molecule HLA-DR and the co-stimulatory molecules B7.1 (CD80) and B7.2 (CD86) by FACS. The antigen presenting cell surface marker HLA-DR present on mature DCs is critical for presenting antigen to T cells and the co-stimulatory molecules B7.1 and B7.2 activate T cells [13-17]. For a two color FACS analysis, cells were stained with CD1a and HLA-DR, CD1a and B7.1, and CD1a and B7.2. Results showed that hES derived DCs are positive for HLA-DR, B7.1, and B7.2 surface expression (Figure 3). The expression levels are comparable between hES-DCs and FL-DCs: CD1a^+^HLA-DR^+ ^(10.5% and 9.6%), CD1a^+^B7.1^+ ^(14% and 15.4%), and CD1a^+^B7.2^+ ^(11.4% and 12.9%) cells. We also observed single positive cell populations for CD1a, HLA-DR, B7.1, and B7.2 which most likely represent other subsets of DCs. These above data indicate that phenotypically normal DCs can be generated from hES-CD34+ cells.

### hES-DC express Dendritic Cell-specific ICAM-3-grabbing nonintegrin (DC-SIGN)

DC-SIGN (CD209) is a DC-specific C-type lectin which is expressed by mature DCs. DC-SIGN plays a vital role in establishing the initial contact between DCs and resting T cells through its recognition of ICAM-3 receptor [13-15]. In addition, with regard to HIV infection, it was found that DC-SIGN bound virus is more stable and is more efficiently transferred to susceptible target cells [13,14]. We therefore examined the hES-DCs for its presence by staining with anti-DC-SIGN-PE and anti-CD1a-PECY5 conjugated antibodies. Results showed that a significant percentage of hES-DC (14.4%) express DC-SIGN similar to FL-DCs (14.3%) (Figure 3). We also observed cell populations single positive for CD1a and DC-SIGN which could represent other DC subtypes.

### hES-DCs are capable of allogeneic T cell stimulation

**Figure 4:**
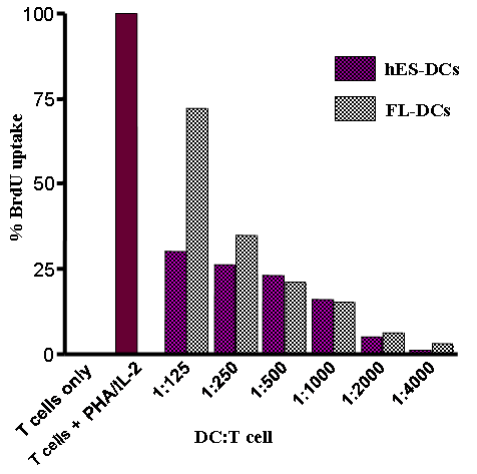
**Allogeneic T-cell stimulation by hES and FL derived DCs**: The allogeneic stimulatory properties of DCs were assessed in a mixed leukocyte reaction assay using allogeneic T-cells. Graded numbers of sorted and irradiated DCs were co-cultured with 5 × 10^5 ^allogeneic T cells. BrdU incorporation was determined by FACS using a PE-conjugated antibody against BrdU. Histograms depict relative percent of BrdU uptake when compared to positive control cells stimulated with IL-2 and PHA. The X-axis is expressed as ratio of stimulator DCs cells to allogeneic responder T cells.

Since DCs are capable of potent stimulation and proliferation of allogeneic T cells, we sought to determine whether the hES-DCs were also able to elicit such a response in a mixed leukocyte reaction (MLR). The day 12 differentiated hES-DCs and FL-DCs, immuno-magnetically sorted based on CD1a^+ ^were used, and proliferation was measured by BrdU uptake using FACS after staining with PE-conjugated anti-BrdU antibody as described in Methods. Results showed that hES-DCs mediated a significant stimulation of allogeneic T cells similar to FL-DCs (Figure 4). The ratio of DCs to the T cells in the reaction mix is expressed as 1:125, 1:250, 1:500, 1:1000, 1:2000 and 1:4000. Highest stimulation was seen with the lower DC to T cell ratio (1:125) and the levels of stimulation decreased at higher ratios. The percent stimulation of T-cells by hES-DCs and FL-DCs were similar at the ratios 1:500 to 1:4000. These results demonstrated the capacity of hES-DCs for allogeneic T cell stimulation.

### hES-DCs are capable of antigen uptake

**Figure 5:**
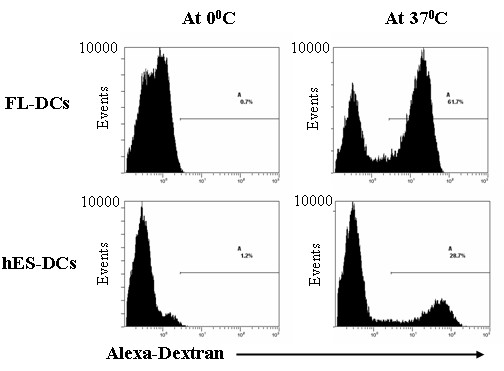
**Antigen uptake by hES-DCs**: Cultured hES and FL DCs were sorted based on CD1a marker. The cells were then incubated with Alexa-Dextran at 0°C and 37°C for 1 hr and analyzed by FACS as described in Methods. The percent antigen uptake was measured as the difference in percentages between the test (37°C) and control (0°C). The percent positive cells are indicated in the plots for both hES-DCs and FL-DCs. Data are representative of triplicate experiments.

An essential function of the DCs is their ability to capture and present antigen to T-cells. The capacity of DCs to take up antigens was measured by using Alexa-dextran, as an indicator of mannose-receptor (MR)-mediated endocytosis. The level of antigen uptake by DCs was expressed as the difference in percentages between the test samples incubated at 37°C versus the controls at 0°C. Results showed that hES-DCs are capable of antigen uptake. However, the levels of uptake were found to be about one fold less than that of FL-DCs (27.5% versus 61.0%) (Figure 5). This could be due to differences in cell types of origin and/or due to their physiological condition at the time of harvest. These results further support the notion that the hES-DCs are functionally competent in addition to being morphologically and phenotypically normal.

### hES-DCs can support productive HIV-1 infection

**Figure 6:**
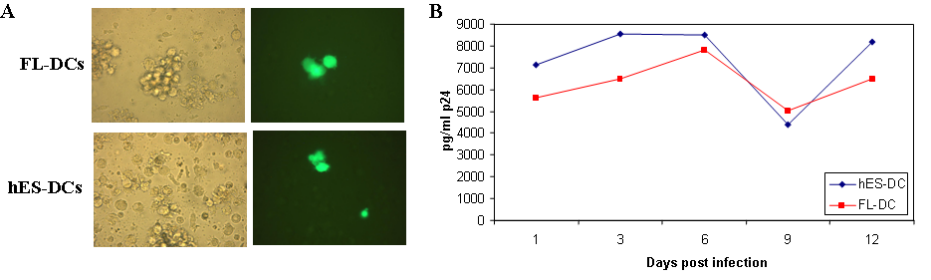
**HIV-1 infection of hES and FL DCs**: To determine virus susceptibility, FL and hES DCs were infected with a replication competent X4-tropic HIV-GFP reporter virus strain at an m.o.i. of 0.2. At 6 days post infection, cells were visualized by fluorescence microscopy to determine GFP expression in productively infected cells at the single cell level. Phase contrast and fluorescence images are shown for the respective cell types (A). Infected culture supernatants were assayed for viral p24 antigen by ELISA at different days post-infection (B). Data is representative of duplicate experiments.

The above results have collectively shown that hES-DCs are similar to normal DCs as demonstrated by comparative analysis with FL-DCs. Apart from being critical for host immunity, DCs can be infected and disabled by viruses such as HIV-1 [13,14]. As mentioned above, DCs also play an important role in the natural history of HIV infection. At the early phase of HIV-1 transmission, DCs capture HIV-1 at mucosal surfaces and transmit the virus to T-cells in the vicinity. Capture of the virus on DCs is known to take place via C-type lectin DC-SIGN surface molecule. Therefore, we wanted to determine if hES-DCs were susceptible to HIV-1 infection as compared to normal DCs. Accordingly, the CD1a-sorted hES-DCs and FL-DCs were exposed to HIV-GFP, a T-tropic virus containing the gene for green fluorescent protein (GFP). Our results showed that both of the virus-exposed hES-DCs and FL-DCs supported viral infection based on GFP expression by the respective cells. However, not all the cells in the culture were productively infected as only a fraction of cells were virus positive for GFP expression. (Figure 6). Supernatants from infected DC cultures were also positive of p24 viral antigen indicative of productive infection. The levels of virus production were not copious however, which is not unexpected since DCs are known to support only a low level viral replication [13,18].

## Discussion

Towards the goal of exploiting hES cells for novel hematopoietic cell reconstitution and HIV gene therapies, here we have shown that phenotypically normal and functionally competent dendritic cells could be differentiated from hES-CD34+ cells. Moreover, we also have demonstrated for the first time that hES-DCs can be productively infected with HIV-1 thus allowing future testing of anti-HIV therapeutic genes such as siRNAs for efficacy in these cells.

In these studies, we induced hES derived CD34+ cells to differentiate into myeloid DCs in the presence of cytokines SCF, GM-CSF, Flt3, IL-3, TNF-α, and IL-4. CD34+ cells derived from human fetal liver were also evaluated in parallel for comparison. Based on FACS analysis for surface markers during culture, CD34+ cells differentiated into mature myeloid DCs showing the typical CD1a phenotype similar to those derived from FL CD34+ cells. The morphology and phenotypic characteristics of hES-DCs were found to be similar to that of DCs derived from fetal liver CD34+ cells cultured in parallel.

It is important that hES-DCs are also functionally normal for future applications. Therefore we analyzed the functional markers HLA-DR (MHC-II), B7.1 (CD80), and B7.2 (CD86) typically expressed by mature DCs. The antigen presenting cell surface marker, HLA-DR present on mature DCs is critical for antigen presentation to CD4^+ ^T cells and the co-stimulatory molecules B7.1 and B7.2 are needed to activate T cells. The mature hES-DCs expressed HLA-DR, B7.1, and B7.2 surface molecules which were comparable with levels expressed in FL-DCs. Consistent with the DCs' ability, the hES-DCs also showed normal capacity for antigen capture as evidenced by dextran uptake. We further analyzed the capacity of hES-DCs to induce proliferation of allogeneic T cells in a mixed leukocyte reaction. Our results showed that hES-DCs indeed are capable of mediating this allogeneic response. We also evaluated the expression of another cell surface molecule, DC-SIGN (CD209) which is a DC-specific adhesion receptor belonging to the C-type lectin family involved in the interactions with T cells [13,14]. Our results showed similar levels of DC-SIGN in hES-DCs and FL-DCs. The above data taken together showed that hES-DCs are phenotypically and functionally normal.

It is known that HIV-1 can infect DCs with the virus remaining stable for long periods. DCs transmit the virus efficiently to CD4 T cells and therefore play an important role in HIV-1 infection. Our results showed that hES-DCs were susceptible to HIV-1 infection similar to that of FL-DCs. However, not all the cells in the culture were productively infected and the levels of viral production are low. This is consistent with previous findings that DCs support only a low level replication and fully mature DCs may have a block in viral replication [13,14]. A recent report has also demonstrated the derivation of physiologically normal DCs from hES-CD34+ cells using a different protocol and compared these to those derived from adult human CD34+ cells and peripheral blood monocytes [19]. Our results are in agreement and confirmed these previous findings, and additionally extended them further by demonstrating that hES-DCs are also susceptible to HIV-1 infection.

In HIV-1 disease, infection of CD4 T cells leads to their eventual decline whereas infection of monocytes/macrophages and dendritic cells leads to continued viral spread and defects in antigenic presentation thus exacerbating the disease process [14]. We previously demonstrated the derivation of macrophagres from hES-CD34+ cells whereas studies of Galic et al [20] derived functional T cells from hES-CD34+ cells in vivo using humanized mice. Whether the hES derived T cells support HIV-1 infection remains to be determined. Lentiviral vector transduction of hES cells and derivation of functional macrophages and T cells that retained the expression of the transgene established that hES cells are tractable for deriving gene modified end-stage primary hematopoietic cells [10,20]. Moreover, our present results together with our previous findings that both hES derived DCs and macrophages are susceptible to HIV infection paved the way for testing anti-HIV constructs introduced into either hES cells or their derivative hematopoietic progenitor CD34+ cells. Thus far many previous studies including our own evaluated a variety of anti-HIV gene constructs in a hematopoietic stem cell setting using CD34+ cells from routine sources such as bone marrow and cord blood [11]. Newer and more potent novel constructs such as siRNAs are currently being investigated some of which are currently entering clinical trials [21]. In addition to anti-HIV genes with a direct inhibitory action on viral molecules, siRNAs and ribozymes that down regulate cellular molecules that aid in HIV-1 infection such as viral coreceptors CCR5 and CXCR4 also show considerable promise [22,23]. Such constructs can now be introduced into hES cells and their efficacy tested in end-stage cells represented by DCs, macrophages, and T cells.

In summary, our data demonstrated the development of terminally differentiated DCs derived from hES cells. The hES-DCs display typical DC morphology, express normal phenotypical markers, are capable of antigenic stimulation, and support HIV-1 infection.

## Conclusion

Phenotypically normal and functionally competent dendritic cells could be derived from hES-CD34+ cells. Large numbers of these hES-DCs cells can now be cultured from a renewable source for use in cell and immune-based therapies. Since these cells also support productive HIV-1 infection, they provide a uniform source of DCs for viral infection studies. It is also now feasible to gene transduce either hES cells themselves and/or hES derived CD34+ cells with anti-HIV genes such as inhibitory siRNAs and test their antiviral efficacy in down stream differentiated DCs which are among the primary target cells that need to be protected against HIV-1 infection.

## Methods

### hES cell growth and propagation

H1 human embryonic stem cell line (hES) was obtained from WiCell (Madison, Wisconsin). The undifferentiated cells were maintained by co-culture with mitomycin C treated mouse embryonic fibroblast (MEF) cells (Chemicon, Temecula, CA) in DMEM/F12 medium supplemented with 20% knockout serum replacer (Invitrogen), 1% MEM-non essential amino acids (Invitrogen), 1 mM L-glutamine, 0.1 mM β-mercaptoethanol (Invitrogen), 0.5% penicillin/streptomycin and 4 ng/ml human basic fibroblast growth factor (Invitrogen). Culture media was replaced daily with fresh complete medium. Mature colonies were subculture weekly by digesting with collagenase IV (Invitrogen) as previously described [10].

### Differentiation of hES cells into DCs

The undifferentiated hES cells (H1) were harvested by treatment with 1 mg/ml collagenase IV (Invitrogen) and dispersed by scraping to maintain the cells in small clumps. The hES cells were added to irradiated (35 Gy) S17 mouse bone marrow derived cell layers and cultured with differentiation media composed of RPMI supplemented with 15% FBS (HyClone), 2 mM L-glutamine, 0.1 mM β-mercaptoethanol, and 1% MEM-nonessential amino acids, 1% penicillin/streptomycin. Media was changed every 2 to 3 days. After indicated days (14–17 days), the differentiated hES cystic bodies were harvested and digested into single cell suspension using collagenase type IV followed by 0.05% trypsin/EDTA supplemented with 2% chick serum (Invitrogen) for 20 minutes at 37°C. Cells were washed twice with phosphate-buffered saline (PBS), filtered through a 70-μM cell strainer (BD Biosciences). To assess the levels of CD34+ hematopoietic progenitor cells in the bulk cell suspension, cells were labeled with PE conjugated anti-CD34+ antibody (BD Biosciences, San Jose, CA) and analyzed by FACS. To purify the CD34+ cells, Direct CD34+ Progenitor Cell Isolation Kit (Miltenyi Biotech, Auburn, CA) was used as recommended by the manufacturer's protocol. Isolated CD34+ cell purity was determined by FACS like above. For comparative experiments, human CD34+ cells were also purified from fetal liver tissue as described above [24]. To derive DCs, the purified CD34+ cells (~4 × 10^5 ^to 6 × 10^5 ^cells) were cultured in Iscove's media containing 10 ng/ml each of SCF, IL-3, TNF-α, IL-4 and 50 ng/ml each of GM-CSF and Flt-3. The differentiated mature dendritic cells were used for subsequent phenotypic and functional analysis.

### Phenotypic analysis of hES-DCs

To determine if hES derived DCs were phenotypically normal, analysis of the characteristic cell surface markers was performed by FACS using respective conjugated antibodies against CD1a-PECY5, CD14-PE, HLA-DR-PE, B7.1-PE, B7.2-PE and DC-SIGN-PE. Fetal liver CD34+ cell derived DCs were also evaluated in parallel. Blocking step was first performed by incubating the cells with the respective isotype sera control for 30 minutes at 4°C before staining with the respective cell surface marker antibodies. Isotype control staining was used to determine background levels. FACS analysis was performed on Beckman-Coulter EPICS^®^XL-MCL flow cytometer with data analysis using EXPO 32 ADC software (Coulter Corporation, Miami, FL). A minimum of 10,000 cells were analyzed in each FACS evaluation.

### Functional analysis of hES-DCs by Mixed Leukocyte Reaction (MLR) assay and antigen uptake assay

The T cell stimulatory capacity of DCs derived from hES cells CD34+ progenitor cells was assessed by co-incubating graded numbers of CD1a^+ ^cells previously sorted on the basis of CD1a immunomagnetic labeling (Miltenyi Biotech, Auburn, CA), and irradiated (3500 rads) DCs for 5 days with 5 × 10^5 ^allogeneic peripheral T cells isolated from peripheral blood using a column purification method to isolate resting T cells per manufacturer's instruction (Cedarlane, Ontario, CA). BrdU (10 μM final concentration) was added 18 hr before harvest and incorporation was measured by permeabilizing the cells with ice cold 70% ethanol for 20 min followed by washing in PBS. The cells were resuspended in freshly prepared 2 N HCl and incubated for 20 min at room temperature to denature nuclear DNA. The cells were then neutralized with 0.2 M disodiumborate and washed with PBS twice. Cells were stained for 20 min with anti-BrdU antibody conjugated with PE (BD-Pharmingen, San Jose, CA). Cells were washed with PBS and analyzed by FACS to determine the percent incorporation of BrdU which is indicative of proliferation. The percent BrdU was determined as a function of input number of sorted DCs and plotted as percent BrdU staining vs. input numbers of DCs. Alexa-dextran was used to assess cell endocytosis as previously described [25]. The antigen uptake capacity was determined using CD1a^+ ^immunomagnetic purified hES-DCs and FL-DCs. Cells resuspended in 10% FBS Iscove's medium (~1 × 10^5 ^cells) were incubated with 1 mg/ml Alexa-dextran at 37°C and 0°C for 60 minutes. The cells were later washed with PBS five times prior to FACS analysis. The level of antigen uptake by DCs was expressed as the difference in percentages between the test (37°C) and control samples (0°C). Fetal liver derived CD34+ cells were also evaluated in parallel.

### HIV-1 infection of hES cells derived dendritic cells

To determine if hES-DCs can be infected with HIV-1 and support viral replication, cells were incubated with a X4 tropic replication competent HIV-GFP reporter virus NLENG-IRES [10,26]. An m.o.i of 0.2 in the presence of 4 μg/ml polybrene was used. Infected cells were visualized by fluorescence microscopy to identify GFP expressing cells. Infected culture supernatants were also assayed for p24 antigen by ELISA using a Coulter-p24 kit (Beckman Coulter, Fullerton, CA).

## Competing interests

The author(s) declare that they have no competing interests.

## Authors' contributions

SB derived the experimental data and RA was responsible for the conception and overall implementation of the project. All authors read and approved the final manuscript.
